# Disturbance–diversity relationships of microbial communities change based on growth substrate

**DOI:** 10.1128/msystems.00887-23

**Published:** 2024-01-23

**Authors:** Don Q. Hoang, Lindsay R. Wilson, Andrew J. Scheftgen, Garret Suen, Cameron R. Currie

**Affiliations:** 1 Department of Bacteriology, University of Wisconsin–Madison, Madison, Wisconsin, USA; 2 Department of Energy Great Lakes Bioenergy Research Center, University of Wisconsin–Madison, Madison, Wisconsin, USA; 3 Microbiology Doctoral Training Program, University of Wisconsin–Madison, Madison, Wisconsin, USA; 4 Department of Pediatrics, University of Pittsburgh, Pittsburgh, Pennsylvania, USA; 5 Department of Biochemistry & Biomedical Sciences, M.G. DeGroote Institute for Infectious Disease Research, McMaster University, Hamilton, Ontario, Canada; Institute for Systems Biology, Seattle, Washington, USA

**Keywords:** disturbance, cellulose, microbial communities, microbial ecology, leaf-cutter ant

## Abstract

Disturbance events can impact ecological community dynamics. Understanding how communities respond to disturbances and how those responses can vary is a challenge in microbial ecology. In this study, we grew a previously enriched specialized microbial community on either cellulose or glucose as a sole carbon source and subjected them to one of five different disturbance regimes of varying frequencies ranging from low to high. Using 16S rRNA gene amplicon sequencing, we show that the community structure is largely driven by substrate, but disturbance frequency affects community composition and successional dynamics. When grown on cellulose, bacteria in the genera *Cellvibrio*, *Lacunisphaera*, and *Asticcacaulis* are the most abundant microbes. However, *Lacunisphaera* is only abundant in the lower disturbance frequency treatments, while *Asticcacaulis* is more abundant in the highest disturbance frequency treatment. When grown on glucose, the most abundant microbes are two *Pseudomonas* sequence variants and a *Cohnella* sequence variant that is only abundant in the highest disturbance frequency treatment. Communities grown on cellulose exhibited a greater range of diversity (1.95–7.33 Hill 1 diversity) that peaks at the intermediate disturbance frequency treatment or one disturbance every 3 days. Communities grown on glucose, however, ranged from 1.63 to 5.19 Hill 1 diversity with peak diversity at the greatest disturbance frequency treatment. These results demonstrate that the dynamics of a microbial community can vary depending on substrate and the disturbance frequency and may potentially explain the variety of diversity–disturbance relationships observed in microbial systems.

**IMPORTANCE:**

A generalizable diversity–disturbance relationship (DDR) of microbial communities remains a contentious topic. Various microbial systems have different DDRs. Rather than finding support or refuting specific DDRs, we investigated the underlying factors that lead to different DDRs. In this study, we measured a cellulose-enriched microbial community’s response to a range of disturbance frequencies from high to low, across two different substrates: cellulose and glucose. We demonstrate that the community displays a unimodal DDR when grown on cellulose and a monotonically increasing DDR when grown on glucose. Our findings suggest that the same community can display different DDRs. These results suggest that the range of DDRs we observe across different microbial systems may be due to the nutritional resources microbial communities can access and the interactions between bacteria and their environment.

## INTRODUCTION

Disturbance ecology investigates foundational questions of how systems and organisms respond to changing environments. Traditionally, disturbances are defined as discrete events that remove biomass directly or indirectly through displacement or mortality (1, 2). Fires, floods, and volcanic eruptions are classic examples of disturbances that change the community composition by directly impacting species or altering the environment (3, 4). Early theoretical consideration of disturbance in community ecology includes the intermediate disturbance hypothesis (IDH), which predicts that the diversity–disturbance relationship (DDR) follows a “hump-backed” or unimodal curve (5). Support for the IDH has been mixed. Experimental measurements of the DDR for different systems have revealed a variety of trends, including both positive and negative monotonic, unimodal, bimodal, and several nonsignificant DDRs (6). Recent frameworks of disturbance theory accommodate vastly different spatiotemporal scales between systems and disentangle disturbance events and impacts (7, 8).

The advent of high-throughput sequencing has widened the scope of questions that microbial ecology can ask, including research that investigates how disturbance impacts microbial communities. Researchers have studied disturbances in several different systems including marine sediment (9), soil bacterial (10) and soil fungal communities (11), and wastewater communities (12). Microbial systems also display a variety of DDRs, which suggests that rather than trying to support or reject specific DDRs, researchers can better understand disturbance ecology by investigating the underlying factors that lead to different DDRs. Given the vast differences in systems between these studies, it is difficult to determine what specific factors lead to differing responses to a disturbance. Although we know that microbial community responses to disturbances can vary, whether the same community can exhibit different responses to the same disturbance and what factors would cause those differences are relatively underexplored. Moreover, understanding what factors influence responses to a disturbance event is important for predictive power in studying microbial communities.

To address this gap in knowledge, we examined the effects of disturbance on a bacterial community enriched from the refuse pile of the leaf-cutter ant *Atta colombica* that had previously been passaged in the lab on minimal media and cellulose by Lewin et al. (13, 14). Leaf-cutter ant refuse piles are composed of discarded plant biomass that has been partially degraded by the ants’ mutualistic fungal cultivar, *Leucoagaricus gongylophorus* (15). Previous work has demonstrated that these refuse piles are enriched with plant biomass-degrading microbes (16, 17). Focusing on bacterial communities derived from leaf-cutter ant refuse piles, Lewin et al. experimentally evolved cellulose-degrading bacterial communities and investigated their compositional dynamics and cellulolytic abilities (13, 14). During each passage, a portion of the community was aliquoted into a new test tube containing fresh minimal media and a new strip of cellulose. These serial transfer events are analogous to disturbance events, as it is a species-independent method of biomass reduction and provides “survivors” with a replenished ecosystem. This method of proxying disturbance through removing cells has been used in other studies (18).

Lewin et al.’s community was enriched on cellulose, a recalcitrant crystal of β-1,4-linked glucose molecules. Cellulose is insoluble in water and must be cleaved by endocellulases (EC 3.2.1.4) and exocellulases (EC 3.2.1.74) into cellobiose. Cellulase genes have limited distribution in bacteria, but β-glucosidases (EC 3.2.1.21), which cleave cellobiose into glucose, are more widespread (19). Thus, cellulolytic and noncellulolytic microbes compete for cellobiose, and these interactions may impact the community’s composition (20, 21). Lewin et al. (14) evaluated the successional dynamics in this microcosm by measuring the relative abundance of 16S rRNA genes every day for a week and found that a *Cellvibrio* operational taxonomic unit (OTU) was more abundant up to 48–72 hours, before other OTUs became more abundant (14). This finding suggests that a cellulose degrader must proliferate and produce cellulases before noncellulolytic opportunists can take advantage of liberated cellobiose or metabolic byproducts.

Our goal was to understand how substrate complexity interacts with disturbance frequency to shape community diversity. We hypothesize that diversity maximizes on a simple substrate (glucose) at higher disturbance frequencies but maximizes on a complex substrate (cellulose) at lower disturbance frequencies. We reasoned that on a simple substrate with low disturbance, competition exclusion would be a stronger driving force for community assembly while more frequent disturbances would disrupt competitive microbes from establishing. Conversely, on complex substrates, the ability to use the substrate would be a more important driving force. Since cellulases are phylogenetically limited in distribution (19), we hypothesize that the bacteria that initially grow will be those that can degrade cellulose similar to what Lewin et al. observed (14). Frequent disturbances should select for bacteria that can directly use cellulose. As cellulose is degraded into cellobiose, those molecules enrich the surrounding media and feed nondegraders. Thus, at infrequent disturbances, nondegraders can grow making the community taxonomically richer.

To test our hypotheses that diversity maximizes on glucose at high disturbance frequencies and maximizes on cellulose at lower disturbance frequencies, we subjected Lewin et al.’s cellulose-enriched community to two substrate treatments: minimal media supplemented with either glucose or cellulose. Each substrate was then subjected to five disturbance frequencies: passage every 1, 2, 3, 5, or 7 days. At the end of their assigned disturbance regime, we expanded the communities into multiple tubes of their respective substrate and destructively sampled over the course of 1 week. We then extracted DNA from these samples for 16S rRNA gene amplicon Illumina-based sequencing. Next, we analyzed these sequences to determine community composition and measured diversity. By comparing the same disturbance frequency between substrate complexities, we can evaluate how community diversity is affected by the interaction between disturbances and resources.

## MATERIALS AND METHODS

### Enrichment on cellulose

The bacterial community used here was previously enriched on cellulose as reported by Lewin et al. (13, 14). Briefly, approximately 3 mg of refuse dump originating from *A. colombica* refuse piles was added to test tubes containing 5 mL of M63 minimal media and a 1 × 10 cm strip of Whatman Grade 1 cellulose filter paper (GE Healthcare Life Sciences, Pittsburgh, PA). As the filter paper was the only carbon source, it selected for a cellulolytic community. The tubes were grown aerobically with shaking at 30°C. Once the filter paper broke, the test tube was vortexed, and 200 µL of the culture was transferred into a new test tube containing fresh M63 media and a new strip of filter paper. This transferring process was repeated each time the filter paper broke. These communities were previously reported at 20 transfers (13) and 60 transfers (14). For this work, we used community “3A” from Lewin et al. (14).

### Media used

M63 minimal media was used for all experiments. One liter contained 61.5 mM potassium phosphate dibasic (Acros, Geel, Belgium), 38.5 mM potassium phosphate monobasic (Acros, Geel, Belgium), 15.1 mM ammonium sulfate (Gibco, Grand Island, NY), 0.5 mL of an iron solution (1 mg/mL iron sulfate in 0.01 M HCl), 1 mL of 1 M magnesium sulfate solution, 1 mL of 1 mg/mL thiamine solution (Acros, Geel, Belgium), and 5 mL of SPV-4 trace element solution (22). This media was modified with different carbon sources, depending upon the treatment. For glucose treatments, 12.5 mL of 40% filter sterilized glucose was added to per liter of M63 media. For cellulose treatments, 5 g of cellulose powder per 1 L of M63 was added. For tubes that used cellulose paper, one strip of 1 × 10 cm Whatman grade 1 cellulose filter paper (GE Healthcare Life Sciences, Pittsburgh, PA) was added to a test tube containing 5 mL of M63 media.

### Disturbance experiment

**Fig 1:**
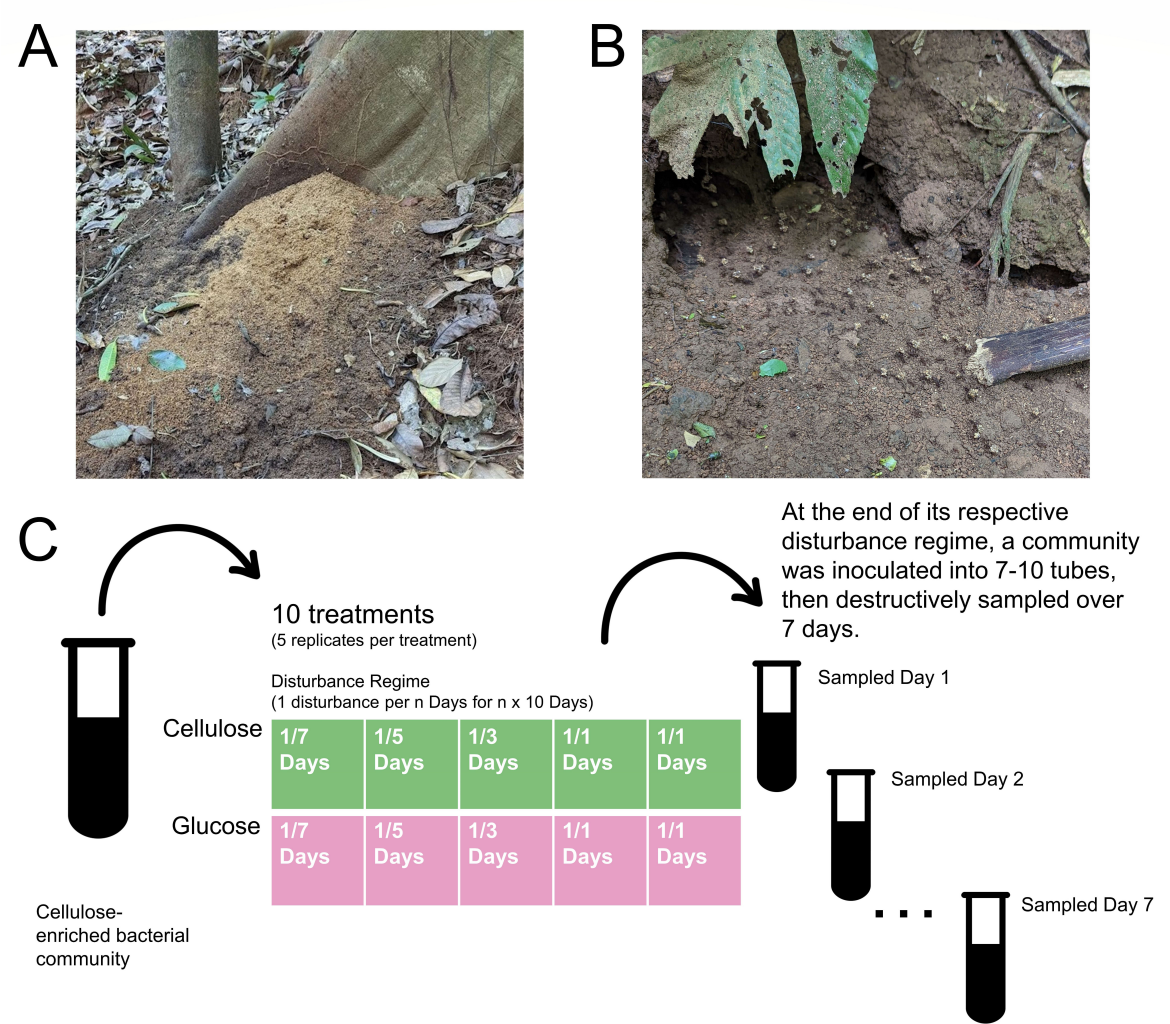
(**A**) Image of *A. colombica* refuse dump. These piles are predominantly composed of partially degraded plant biomass, removed from the bottom of fungus gardens by worker ants. (**B**) Worker ants carrying pieces of refuse material. (**C**) Experimental setup. The cellulose-enriched community was used as the starter inoculum for the growth of microbial communities exposed to 10 treatments. Samples were grown in either cellulose- or glucose-supplemented M63 minimal media, then subjected to one of five disturbance regimes. At the end of their respective disturbance regime, communities were used to inoculate 7–10 tubes (containing their respective substrate), and those tubes were destructively sampled every day for 7 days. “Test tube” icon by art shop and “arrow” icon by Yoteyo, from thenounproject.com CC BY 3.0.

To investigate how substrate complexity and disturbance frequency interact to shape community dynamics, we grew Lewin et al.’s cellulose-enriched community from a freezer stock of transfer #73 in M63 media supplemented with cellulose filter paper with shaking at 30°C. Once the community broke the cellulose filter paper, it was scaled up into a 250-mL culture of M63 media and cellulose filter paper. After 4 days of growth (when we observed visible cellulose paper degradation), the community was split into test tubes representing 10 different treatments (Fig. 1). Communities were grown in 5 mL of M63 minimal media supplemented with either glucose or cellulose. These two media treatments were further divided into five disturbance treatments: every 1, 2, 3, 5, or 7 days, test tubes were vortexed to homogenize the community, and 200 µL of the culture was used to inoculate a new tube of fresh media. Each treatment had five technical replicates. Each disturbance was carried out 10 times. That is, tubes in the 1-day disturbance treatment were transferred every day for 10 days, and tubes in the 7-day disturbance treatment were transferred every week for 10 weeks. We refer to these disturbance treatments as 1/*n* days. For example, treatments that were passaged every 3 days will be referred to as “1/3 days,” indicating one disturbance per 3 days. At the end of their respective disturbance regime, each community was used to inoculate 7–10 tubes, which were destructively sampled every day for 1 week to evaluate any compositional dynamics. Those tubes were then destructively sampled each day for 1 week. At sampling, the bacterial culture was spun down, and the pellet and supernatant were separated. Cell pellets were resuspended in Zymo DNA/RNA shield (Zymo Research, Irvine, CA, USA) and stored at −80°C until DNA extraction.

### DNA extraction and sequencing

We extracted DNA for 16S rRNA gene amplicon sequencing. Samples were extracted using QIAGEN DNeasy PowerSoil kits (QIAGEN, Hilden, Germany) following the manufacturer’s instructions.

For 16S rRNA gene-based community profiling, the V4 regions of the 16S rRNA gene were amplified using bacteria-specific primers (515F: GTGCCAGCMGCCGCGGTAA and 806R: GGACTACHVGGGTWTCTAAT) (23). Each reaction contained 50 ng DNA, 0.4 µM forward primer, 0.4 µM reverse primer, 12.5 µL 2× HotStart ReadyMix (KAPA Biosystems, Wilmington, MA, USA), and water to a final volume of 25 µL. Polymerase chain reaction (PCR) was performed using a Bio-Rad S1000 thermocycler (Bio-Rad Laboratories, Hercules, CA, USA). Cycling conditions began with an initial denaturation at 95°C for 3 minutes, followed by 25 cycles of 95°C for 30 seconds, 55°C for 30 seconds, and 72°C for 30 seconds, and a final extension at 72°C for 5 minutes. We included controls using sterile water in place of DNA to ensure there was no contamination during PCR. PCR products were purified using gel extraction from a 1.0% low-melt agarose gel (National Diagnostics, Atlanta, GA, USA) with a ZR-96 Zymoclean DNA Recovery Kit (Zymo Research, Irvine, CA, USA), and DNA was quantified using a Qubit Fluorometer and Qubit Kit (Invitrogen, Carlsbad, CA, USA). Samples were equimolarly pooled with 10% PhiX control DNA and sequenced on an Illumina MiSeq using a MiSeq 2  ×  250 v2 kit (Illumina, Inc.). Due to the number of samples, sequencing was performed across two sequencing runs.

### Sequence analysis and statistical analysis

Reads were processed using DADA2 (24) in R version 4.2.1 (25) with taxonomy assignment using the SILVA V138.1 reference database (26, 27). Sequencing yielded 11,670,039 sequences, and denoising, merging, and removal of chimeric sequences resulted in 10,499,700 sequences. Samples had an average of 29,410.92 ± 33,930.7 sequences and an average of 18.41 ± 12.62 amplicon sequence variants (ASVs. Of the 357 samples we sequenced, 302 were used for analysis. Using the R package phyloseq (28, 29), we removed negative control samples, samples with low reads (less than 1,000 reads), and five samples that we suspect were mislabeled (Fig. S1). We also used phyloseq to transform ASV read count tables to relative abundance tables prior to downstream analysis. We calculated Hill diversities based on relative abundances to measure the diversity within our samples (30–33). We calculated Hill diversity numbers 0, 1, and 2 using the R package hillR (34). Hill diversity number 0 does not weight “species” abundance and represents the richness or number of ASVs; Hill diversity number 1 weights abundance for all “species,” which is the exponential of Shannon’s entropy or number of common species; and Hill diversity number 2 gives more weight to common “species,” which is the inverse of Simpson’s diversity index or number of very common species (30, 34). We used Student’s *t*-test to determine whether disturbance frequency 1/3 days had the greatest Hill 1 diversity for cellulose treatments and whether disturbance frequency 1/1 days had the greatest Hill 1 diversity for glucose treatments. To evaluate whether a linear curve or unimodal curve better fits our diversity–disturbance data, we performed both a linear regression and a quadratic regression of Hill 1 diversity measurements.

The R package vegan (35, 36) was used for beta-diversity analysis, and we supplemented this analysis with the R package usedist (https://github.com/kylebittinger/usedist). To quantify the differences between our samples, we calculated Bray–Curtis distances of ASV composition in our samples. To test whether there is more similarity within our variable groups (substrate, disturbance frequency, replicate, time sampled, and Illumina run) than between variable groups, we used the R package vegan (35, 36) to perform an analysis of similarity (ANOSIM). To test whether the centers (centroids) of our data clusters significantly differed, we performed a Permutational analysis of variance (PERMANOVA) using the adonis() function in the R package vegan. To evaluate the differences in community variation based on disturbance frequency or time within each substrate treatment, we used the R package usedist (https://github.com/kylebittinger/usedist) to measure the distance between cluster centroids and the distance from samples to cluster centroids. We used Student’s *t*-test to test whether the differences in ordination distance between substrate treatments were statistically significant. Additionally, we used an analysis of variance (ANOVA) to test whether the means of different disturbance frequency treatments or time sampled (within each substrate treatment) were significantly different. Scripts for read processing, diversity analysis, and figure generation can be found at https://github.com/donnyhoang/cellulose_disturbance.

## RESULTS

### Community composition varies between treatments

**Fig 2:**
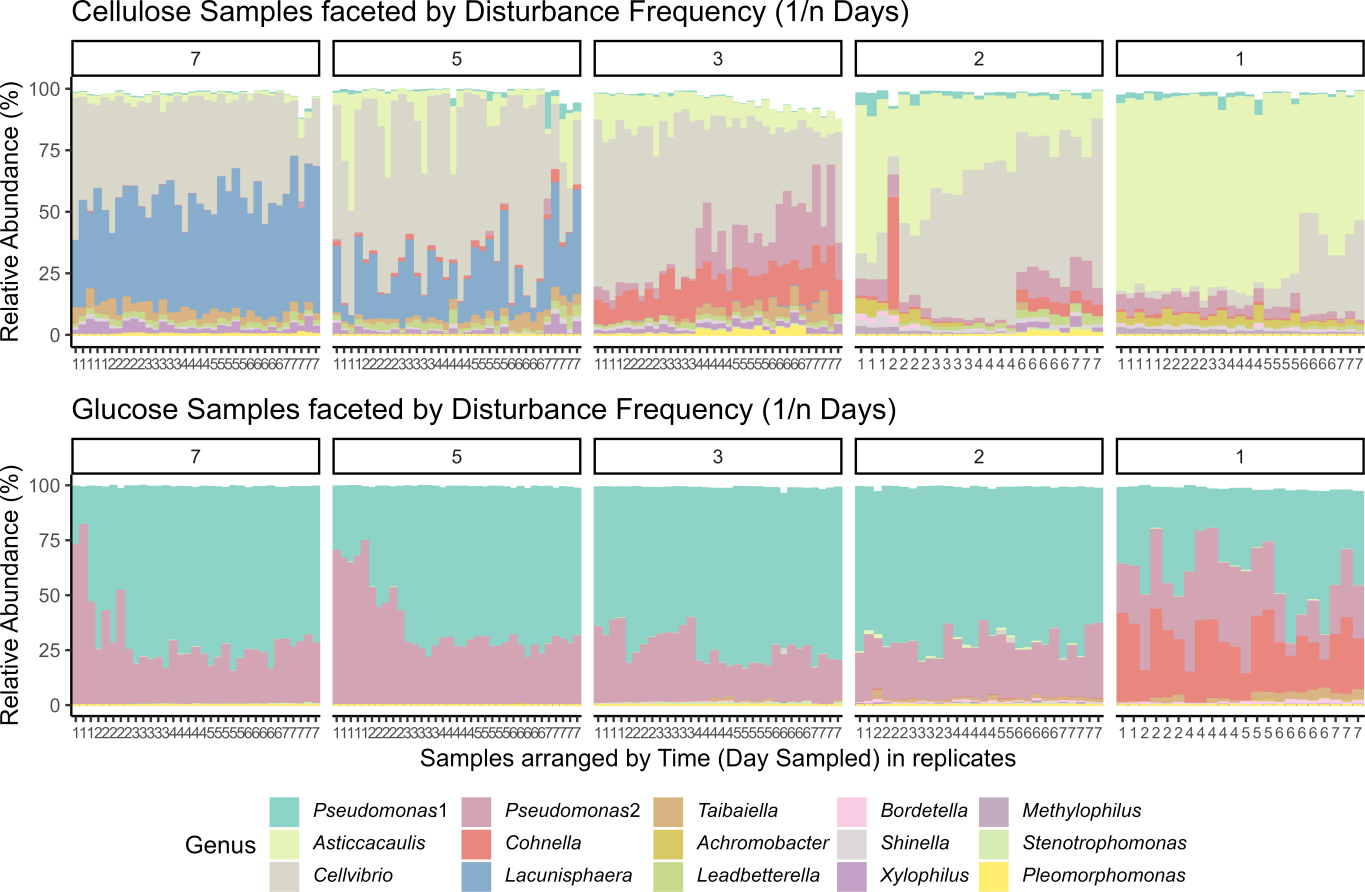
Bar graph of community composition based on relative abundance of 16S rRNA gene amplicon sequencing of the top 15 most abundant ASVs and their assigned bacterial genus. The top row of plots represents samples grown in M63 minimal media and cellulose, and the bottom row of plots represents samples grown in M63 minimal media and glucose. Plots are faceted by disturbance frequency (1/*n* days). Within each facet, samples are grouped by time sampled to observe any compositional dynamics.

Communities grown on different substrates were found to be dominated by different microbes (Fig. 2). Across those treatments with cellulose as the only carbon source, the most abundant microbes include *Cellvibrio*, *Lacunisphaera*, and *Asticcacaulis*, while two *Pseudomonas* ASVs dominated samples with glucose as the only carbon source. However, they were not as abundant in cellulose samples, where they range 0%–14.9% and 0%–32.89% relative abundance. Similarly, the three most abundant ASVs in our cellulose samples were not abundant across the glucose samples. *Cellvibrio* (range 0%–2.71%), *Lacunisphaera* (range 0%–0.5%), and *Asticcacaulis* (range 0%–1.83%) are all low relative abundance in glucose samples.

Disturbance frequency also impacted community composition when communities are grown on cellulose. In the cellulose substrate treatments, communities were dominated by *Asticcacaulis* (range 47.97%–83.05% relative abundance) in the highest disturbance frequency (1/1 days). *Lacunisphaera* (range 0%–0.11%) and *Cellvibrio* (1.16%–42.06%) were present at lower relative abundance in the highest disturbance frequency treatment (1/1 days). At the lowest disturbance frequency (1/7 days), the community was dominated by *Lacunisphaera* (range 26.54%–59.7%) and *Cellvibrio* (18.45%–57.75%) while *Asticcacaulis* was lower (0.49%–7.85%) in relative abundance.

Disturbance frequency does not impact the community composition of our glucose samples as strongly as it did in the cellulose treatment. Two *Pseudomonas* ASVs dominate nearly all the glucose samples. One *Pseudomonas* ASV ranges from 17.03% to 84.25%, and a second *Pseudomonas* ASV ranges 5.63%–82.04% relative abundance. A third ASV belonging to the genus *Cohnella* becomes abundant in the highest disturbance frequency treatment (1/1 days) ranging from 12.6% to 40.49% relative abundance. In other disturbance frequency treatments of glucose samples, *Cohnella* ranges from 0% to 0.66% relative abundance.

Disturbance frequency also impacts community dynamics. When cellulose samples were grown in more frequent disturbance treatments, 1/3 days, 1/2 days, and 1/1 days, there was a clear change over time in community composition. In the 1/3 frequency treatment, *Cellvibrio* was initially abundant (range 60.18%–67.97% on Day 1 to 12.53%–44.99% on Day 7) but gives way to the second *Pseudomonas* ASV and *Cohnella* (14.82%–32.89% on Day 7 and 10.95%–27.71% on Day 7, respectively). In the 1/2 days frequency treatment, *Asticcacaulis* is initially abundant (range 54.3%–60.27% on Day 1 to 11.28%–23.74% on Day 7) before *Cellvibrio* increases in abundance as the week progresses (6.87%–18.85% on Day 1 to 42.07%–68.85% on Day 7). The 1/1 day frequency treatment showed a similar trend to the 1/2 days frequency treatment, but *Asticcacaulis* was dominant across the week of sampling (range 74.61%–80.45% on Day 1 to 52.56%–66.41% on Day 7), and *Cellvibrio* starts low but increases in abundance (1.42%–3.79% on Day 1 to 25.6%–40.43% on Day 7). The cellulose substrate samples subjected to lower disturbance frequency treatments (1/7 days and 1/5 days) did not have a clear successional pattern.

Glucose samples largely did not show a change in community composition across the week we sampled, with most of the community dominated by two *Pseudomonas* ASVs. However, the disturbance frequency treatments 1/7 days and 1/5 days showed some dynamics. In the 1/7 days disturbance frequency treatment, one *Pseudomonas* ASV increases (range 17.03%–52.78% on Day 1 to 67.46%–72.91% on Day and "24.52%–34.79% on Day 1 to 67.03%–71.66% on Day 7") while a second *Pseudomonas* ASV decreases (46.67%–82.0% on Day 1 to 25.93%–30.64% on Day 7) in relative abundance. The 1/5 days treatment showed a similar trend where one *Pseudomonas* ASV increases (24.52%–34.79% on Day 1 to 67.03%–71.66% on Day) and the second *Pseudomonas* ASV decreases (64.41%–74.53% on Day 1 to 27.42%–31.06% on Day 7).

### Communities cluster by growth substrate

**Fig 3:**
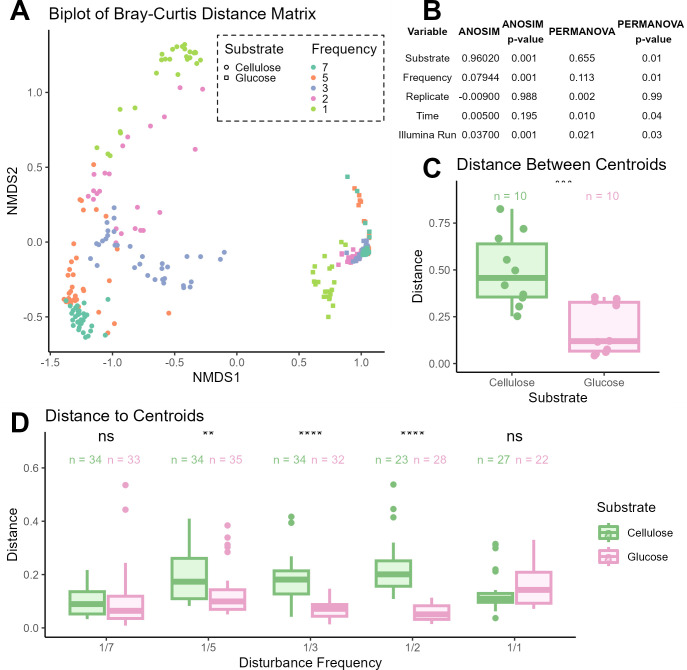
(**A**) NMDS plot of community composition. The distance matrix was calculated using the Bray–Curtis distance method. We examined the first three dimensions in ordination analysis (*k* = 3, stress = 0.037) but visualize a biplot for ease. A 3D view can be found in Fig. S3. Communities grown in cellulose are shown as circles, and communities grown in glucose are shown as squares. Disturbance frequency is marked by color. (**B**) Table of ANOSIM and PERMANOVA calculations of recorded factors that may contribute to variance. (**C**) Boxplot of distances between centroids of data clusters based on disturbance frequency and accounting for substrate treatment (cellulose in green, glucose in pink). The significance label represents Student’s *t*-test, comparing the mean distance between cellulose and glucose treatments. (**D**) Boxplot of distance to centroids of samples, clustered based on disturbance frequency and accounting for substrate. Significance labels represent Student’s *t*-test, comparing the mean distance between cellulose and glucose treatments of the same disturbance frequency. The mean distance between disturbance frequency treatments for cellulose samples (ANOVA *P*-value = 2.0*e*−07) and glucose samples (ANOVA *P*-value = 1.9*e*−05) was statistically significant.

To better quantify the differences between our samples, we calculated Bray–Curtis distances between samples. We examined the first three latent variables in ordination analysis but visualized a biplot for ease of viewing (Fig. 3A; Fig. S3 contain a view of the three-dimensional plot). To test whether there is a significant difference between sample groups, we performed an analysis of similarity. We found that samples were most dissimilar based on substrate (ANOSIM *R* = 0.912, *P*-value = 0.001). Samples separate along the NMDS1 axis based on substrate and form two distinct clusters. Additionally, samples grown on cellulose have greater distances between centroids compared to glucose samples (Student’s *t*-test, *P*-value = 0.00062) (Fig. 3C). Within the two substrate clusters, samples appear to group by disturbance frequency treatment. We measured the distance from each sample to their centroid (when clusters are based on disturbance frequency and substrate). There was no significant difference between the cellulose and glucose samples for the lowest and highest disturbance frequency treatments (Fig. 3D). However, for disturbance frequency treatments 1/5 days, 1/3 days, and 1/2 days, cellulose samples had greater distance to their respective centroids than glucose samples (Student’s *t*-test, *P*-value = 0.0022, *P*-value = 1.0*e*−08, and *P*-value = 1.7*e*−07, respectively). Finally, the means of sample distance to centroid between disturbance frequency treatments was statistically significant for both cellulose treatments (ANOVA *P*-value = 2.0*e*−07) and glucose treatments (ANOVA *P*-value = 1.9*e*−05). Across days sampled, cellulose communities always had greater distance to centroids than glucose samples (Fig. S5). Here, cellulose samples did not differ significantly in the mean of sample distance to centroid (ANOVA, *P*-value = 0.085), but glucose samples did (ANOVA, *P*-value = 2.6*e*−05).

### Diversity–disturbance relationships differ based on substrate

**Fig 4:**
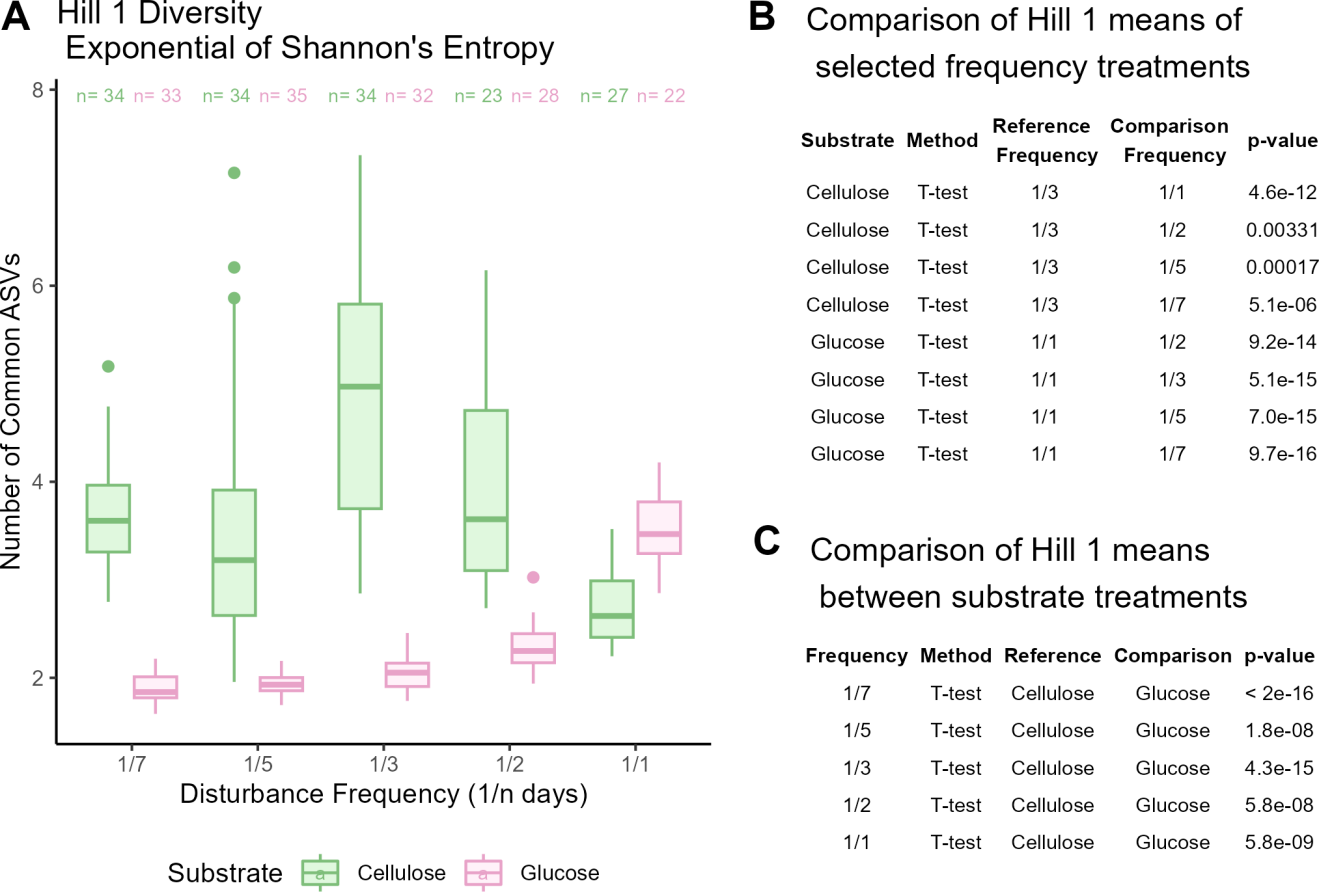
(**A**) Boxplot of Hill 1 diversity. Green boxes represent samples grown on cellulose, pink boxes represent samples grown on glucose, and “*n*” refers to the number of samples within that disturbance frequency treatment. (**B**) Table of Student’s *t*-tests demonstrating that the 1/3 days disturbance frequency for cellulose has the highest mean Hill 1 diversity compared to other cellulose samples and that the 1/1 day disturbance frequency for glucose has the highest mean Hill 1 diversity compared to other glucose samples. (**C**) Table of Student’s *t*-tests comparing means of Hill 1 diversity measurements between cellulose and glucose samples of different disturbance frequency treatments.

To evaluate how substrate complexity and disturbance frequency interact to affect community diversity, we measured the diversity of our samples using Hill diversities (30, 31, 33). We found that diversity changes across disturbance frequency, but this pattern differs depending on the substrate the community was grown in (Fig. 4A). Samples grown in cellulose had the greatest Hill 1 diversity for 1/3 disturbance frequency (mean 4.866 ± 1.22 common ASVs), and this was statistically significant when compared to other disturbance frequency treatments grown on cellulose (Fig. 4B). Samples grown in glucose had the greatest Hill 1 diversity at the highest disturbance frequency (3.51 ± 0.4 common ASVs in the 1/1 disturbance frequency treatment), and this was statistically significant when compared to other disturbance frequency treatments grown on glucose (Fig. 4B).

We also measured Hill 0 (richness) and Hill 2 (inverse Simpson index) (Fig. S3). We observed greater mean Hill 0 diversity for cellulose samples in nearly all disturbance frequencies, except for 1/2 days where there is a nonsignificant difference (Fig. S2A). Cellulose samples have greater mean Hill 2 diversity than glucose samples in all disturbance frequencies, except for the highest disturbance frequency treatment, 1/1 day (Fig. S2B).

We performed both a linear regression and quadratic regression to test for a unimodal or monotonic pattern of the Hill 1 diversity and disturbance frequency relationship. We found that neither a linear nor quadratic shape fits the cellulose data (Fig. S4). Adding a quadratic term improves the fit (linear regression *R*^2^ = 0.10; quadratic regression *R*^2^ = 0.25). A linear model better describes the glucose Hill 1 diversity data (*R*^2^ = 0.84), and adding another term improves the fit (*R*^2^ = 0.87).

## DISCUSSION

In this study, we aimed to address how substrate and disturbance frequency interact to shape microbial community structure. We found that substrate is a main driver of the communities’ response to disturbance. We demonstrated that diversity peaks at the intermediate disturbance frequency, 1/3 days, when the community is grown on cellulose, a recalcitrant carbon source. However, community diversity peaks at the highest disturbance frequency, 1/1 day, when the community is grown on glucose, a labile carbon source. The results of this work show how community response to disturbances can be impacted by the substrate they are grown in and contribute to our understanding of how environmental factors interact with disturbances to impact bacterial communities.

### Diversity–disturbance relationships

Our community displays a different DDR depending on the substrate it is grown on. When grown on cellulose, the diversity peaks at an intermediate disturbance frequency (Fig. 4) that fits with predictions of the intermediate disturbance hypothesis, which posits that diversity peaks at intermediate disturbances (5). However, the IDH has been found to be an inadequate framework as studies across a variety of ecosystems have found many divergent types of DDRs (6, 37). Our findings also demonstrate the incompleteness of the IDH; communities grown on glucose peak in diversity at our highest disturbance frequency treatment. Differing DDRs resulting from the same experimental system have been observed before. Hall et al. (18) manipulated disturbance intensity (the proportion of cells they moved) and used a simpler one-species community—exploiting the ability of *Pseudomonas fluorescens* to exhibit distinct morphotypes based on access to oxygen. They found a flat, monotonically increasing, or unimodal DDR depending on the disturbance intensity (18).

We ran model fitting to test for a unimodal or monotonic pattern for our Hill 1 diversity measurements but found that neither a linear nor a quadratic regression fits our cellulose samples (Fig. S4). A linear regression led to a poor model fit (linear regression, *R*^2^ = 0.10). Adding another term improved the fit (quadratic regression *R*^2^ = 0.25) but was still poor overall as only 25% of the variance in our measured Hill 1 diversity is explained by the model. Additionally, plotting the residuals of these models reveals nonrandom patterns (Fig. S4C). Both linear and quadratic models for cellulose samples typically have positive values while both linear and quadratic models for glucose samples typically have negative residuals. We speculate that the poor fit, especially for our cellulose samples, is due to our longitudinal sampling where bacterial ASV abundances are changing over time. A model that incorporates time may better fit our data. Since our Student’s *t*-test demonstrates that diversity peaks at the intermediate disturbance frequency (1/3 days) for cellulose samples and that diversity peaks at the greatest disturbance frequency (1/1 day) for glucose samples, we conclude that samples grown on cellulose have a unimodal DDR while samples grown on glucose have a monotonically increasing DDR.

Other experiments have found a variety of DDRs, including a U-shaped DDR (38). A model of a two-member community, based on experimental observations, consistently found unimodal DDR, although the exact shape changed with time (39). A more recent model of a two-member community displayed multimodality (40). One potential reason we did not observe a unimodal DDR with our glucose treatment could be because we did not have a disturbance regime that was frequent enough to result in a population bottleneck. If the disturbances were so frequent that no, or very few, microbes were being passaged each time, then we might expect the diversity of the glucose communities to decrease.

### Extent of variation and substrate

Cellulose samples displayed greater variation between samples than glucose samples (Fig. 3A and C). Furthermore, within substrate treatments, disturbance frequency affects community variation. These findings reflect ASV compositional patterns within each treatment (Fig. 2). Additionally, we used an ANOVA to test whether there was a significant difference between the mean distance to centroid between disturbance frequency treatments (Fig. 3D). We found that both cellulose and glucose samples were significantly different across disturbance frequencies. These findings suggest that disturbance frequency affects the variation, but the extent of this variation depends on the substrates. We performed a similar analysis but grouped our samples by time rather than disturbance frequency (after accounting for substrate) (Fig. S5). The mean distances of cellulose samples are always greater than those of glucose samples across time, but cellulose samples are not significantly different from each other. The mean distance of glucose samples, however, is significantly different from each other. This finding suggests that even though time may explain some variation, substrate is still the primary factor for variation.

### Disturbance disrupts community composition

Following their assigned disturbance regime treatment, we sampled our experimental communities over the course of a week to evaluate how disturbance frequencies may impact community assembly. In the intermediate disturbance frequency for cellulose treatment (1/3 days), *Cellvibrio* is typically abundant, before being replaced by other taxa. This succession resembles what Lewin et al. (14) reported. However, at lower frequencies (1/7 and 1/5 days), *Lacunisphaera* was also found to be abundant, and at higher frequencies (1/2 days and 1/1 days), *Asticcacaulis* increases in relative abundance. Notably, *Cellvibrio* starts at lower relative abundance before increasing in the high-frequency disturbance treatments (1/2 and 1/3 days).

It is important to note that *Lacunisphaera* was not identified in Lewin et al.’s work. *Lacunisphaera* spp., which belong to the Verrucomicrobia phylum, do not have any reported cellulolytic activity, although one isolate has been described to use a variety of carbon sources (41). An *Asticcacaulis* ASV was abundant in our high-frequency cellulose treatments, and an *Asticcacaulis* OTU was found in (13). *Asticcacaulis* has been found in other lignocellulolytic communities including communities derived from wood or forest soil (42, 43).

Communities grown in glucose did not display obvious assembly patterns at most disturbance frequencies. We identified two abundant *Pseudomonas* ASVs in the glucose substrate treatments. We cannot determine if these represent different populations, but they appear to have different dynamics across disturbance frequencies. As our study was limited to 16S rRNA gene amplicon sequencing, we cannot determine what mechanisms led to the abundance of *Pseudomonas* ASVs in the glucose samples. *Pseudomonas* is a common environmental microbe, known best as a soil-dweller or member of the rhizosphere microbiome (44). As enteric bacteria, the *Pseudomonas* ASVs likely have faster growth rates than other ASVs in these communities. Enrichment for copiotrophs when growth substrate is supplemented with labile carbon has been observed in a previous study (45). Additionally, *Pseudomonas* are known for producing a variety of natural products, including molecules that suppress competing microbes (46, 47). This may be one explanation for how it came to dominate the glucose samples.

The two *Pseudomonas* ASVs dominated community composition in most disturbance frequencies for communities grown in glucose, except for the highest frequency treatment, which also had highly abundant *Cohnella* ASV. An isolate from this genus has shown cellulolytic ability (48, 49). Although we cannot explain why it is abundant in the high-frequency glucose samples, the same ASV is also found in the intermediate frequency of our cellulose samples, which matches the report of Lewin et al. that found *Cohnella* to be positively associated with cellulose degradation (14). They did not recover a metagenome-assembled genome classified to *Cohnella*.

The different diversity peaks and successional patterns we observe are likely due to the differing interactions between microbes in the two substrates we considered. Cellulose is a recalcitrant substrate that must be cleaved into cellobiose (a glucose dimer), which is transported into the cell before being cleaved into glucose (50). *Cellvibrio* is likely the dominant cellulose degrader in this microcosm (13, 14). In order to degrade cellulose, *Cellvibrio* produces extracellular endoglucanases (EC 3.2.1.4) and exoglucanases (EC 3.2.1.74) that liberate cellobiose from the cellulose polymer (51). Excess cellobiose molecules are likely what feeds the remaining community. Thus, noncellulolytic organisms cannot immediately consume carbon in our cellulose treatments and must wait for cellulose degraders to enrich the media with labile cellobiose. In contrast, glucose is labile, and thus, competition is likely a much stronger driving force in community dynamics. This study is limited by our choice to use glucose as a substrate. Cellobiose is more reflective of natural processes, and the insights from this study would be improved had we used it. However, we do not expect dramatically different conclusions, as cellobiose is still labile relative to cellulose.

Given that we used 16S rRNA gene amplicon sequencing, we cannot make definitive conclusions about the type of interactions in our community. Substrate can influence bacterial interactions. For example, one study demonstrated that synergistic interactions found in co-cultures of *Citrobacter freundii* and *Sphingobacterium multivorum* on carboxymethyl cellulose, xylan, and lignin or wheat straw were lost when the pair was grown on glucose (52). We cannot make a direct comparison, since cellulose is only structurally complex compared to glucose (a polysaccharide compared to a monosaccharide), while xylan and lignin are more complex in other ways. For example, the xylan backbone is a polymer of xylose but is decorated with a diversity of side groups (i.e., acetate, uronic acid, and ferulic acid), and xylose groups can be substituted with arabinose. These side groups can form linkages to other xylan chains or lignin, and the deconstruction of xylan involves both the removal of these side groups and depolymerization of the polysaccharide chain (53). A future study could investigate how the complexity of other lignocellulose biomass influences the response of a community to disturbance frequency.

### Conclusion

Here, we have demonstrated that communities will respond differently to the same disturbance regime, when grown on substrates of varying complexity. We observe a peak in diversity at an intermediate disturbance frequency when communities are grown on cellulose, a recalcitrant substrate. When grown on glucose, however, we observed a peak in diversity at the highest disturbance frequency. Although substrate is a strong predictor for community composition, communities further cluster by disturbance frequency, and successional dynamics differ between disturbance treatments for the same substrate. These results suggest that the range of DDRs we observe across different microbial systems may be due to the nutritional resources microbial communities can access and the interactions between bacteria and their environment.

## Data Availability

Amplicon sequencing data have been uploaded to the NCBI databases under BioProject number PRJNA1008240. Data Set S1 contains individual accession numbers and relevant metadata for each sample.

## AFTER EPUB

*[This article was published on 23 January 2024 with errors in the Acknowledgments. The Acknowledgments were corrected in the current version, posted on 26 January 2024.]*

## SUPPLEMENTAL MATERIAL

Data Set S1 msystems.00887-23-s0001.xlsx NCBI accession numbers and metadata of 16S rRNA gene sequencing data generated for this work.

Figure S1 msystems.00887-23-s0002.tiff Relative abundance plot of samples from the disturbance frequency 1/2 treatment.

Figure S2 msystems.00887-23-s0003.tiff Boxplot of Hill 0 and Hill 2 diversities.

Figure S3 msystems.00887-23-s0004.tiff A view of a 3D NMDS plot of community composition.

Figure S4 msystems.00887-23-s0005.tiff A revisualization of Fig. 4.

Figure S5 msystems.00887-23-s0006.tiff Boxplot of distance to centroids of samples.

Supplemental legends msystems.00887-23-s0007.docx Legends for supplemental files

ASM does not own the copyrights to Supplemental Material that may be linked to, or accessed through, an article. The authors have granted ASM a non-exclusive, world-wide license to publish the Supplemental Material files. Please contact the corresponding author directly for reuse.
