# Supplementary figures and images for: Disturbance–diversity relationships of microbial communities change based on growth substrate
Source: mSystems. 2024 Jan 23;9(2):e00887-23. doi: 10.1128/msystems.00887-23 (PMC10878081; doi:10.1128/msystems.00887-23)

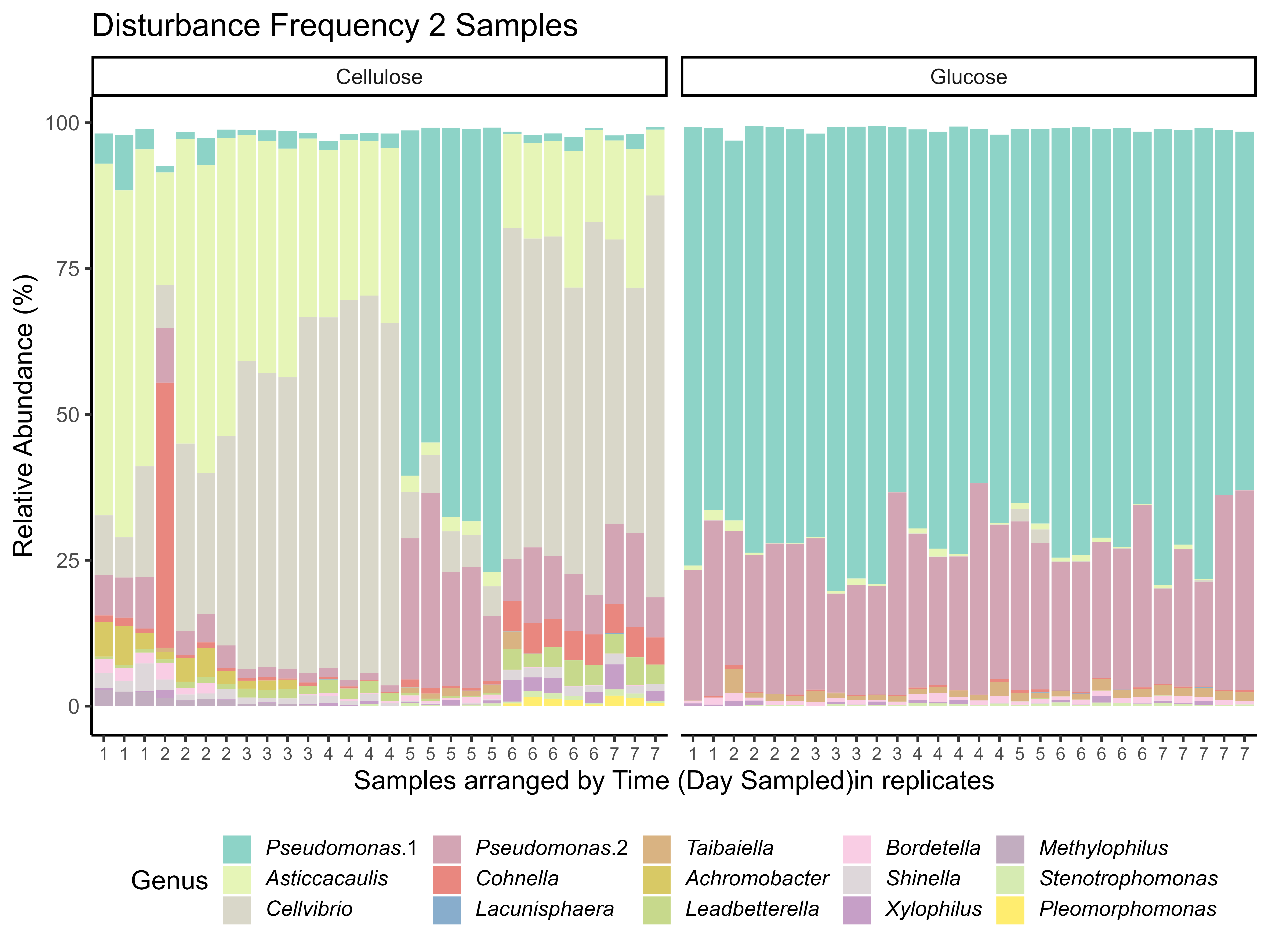

Supplement: Figure S1 — Relative abundance plot of samples from the disturbance frequency 1/2 treatment. [file msystems.00887-23-s0002.tiff]

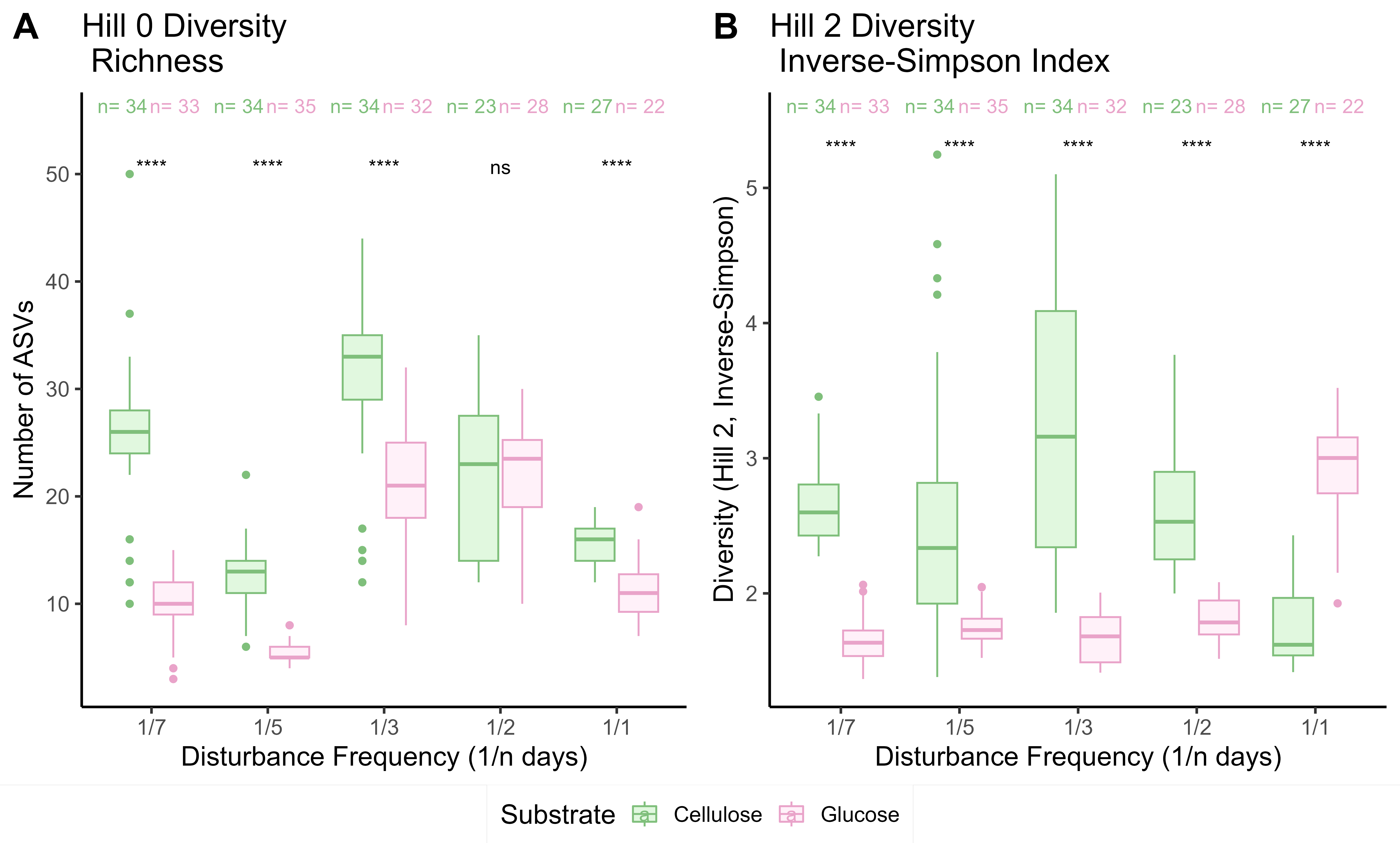

Supplement: Figure S2 — Boxplot of Hill 0 and Hill 2 diversities. [file msystems.00887-23-s0003.tiff]

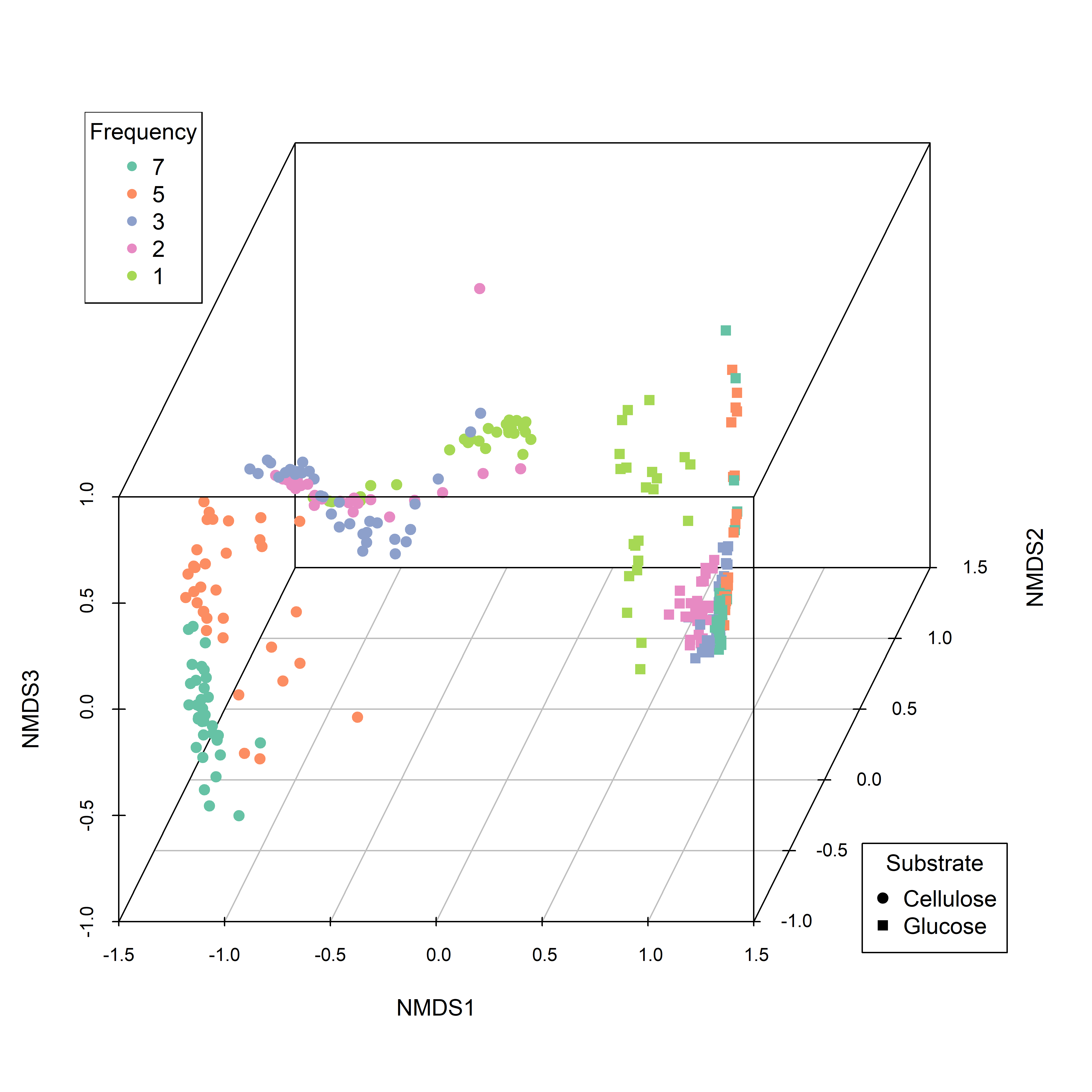

Supplement: Figure S3 — A view of a 3D NMDS plot of community composition. [file msystems.00887-23-s0004.tiff]

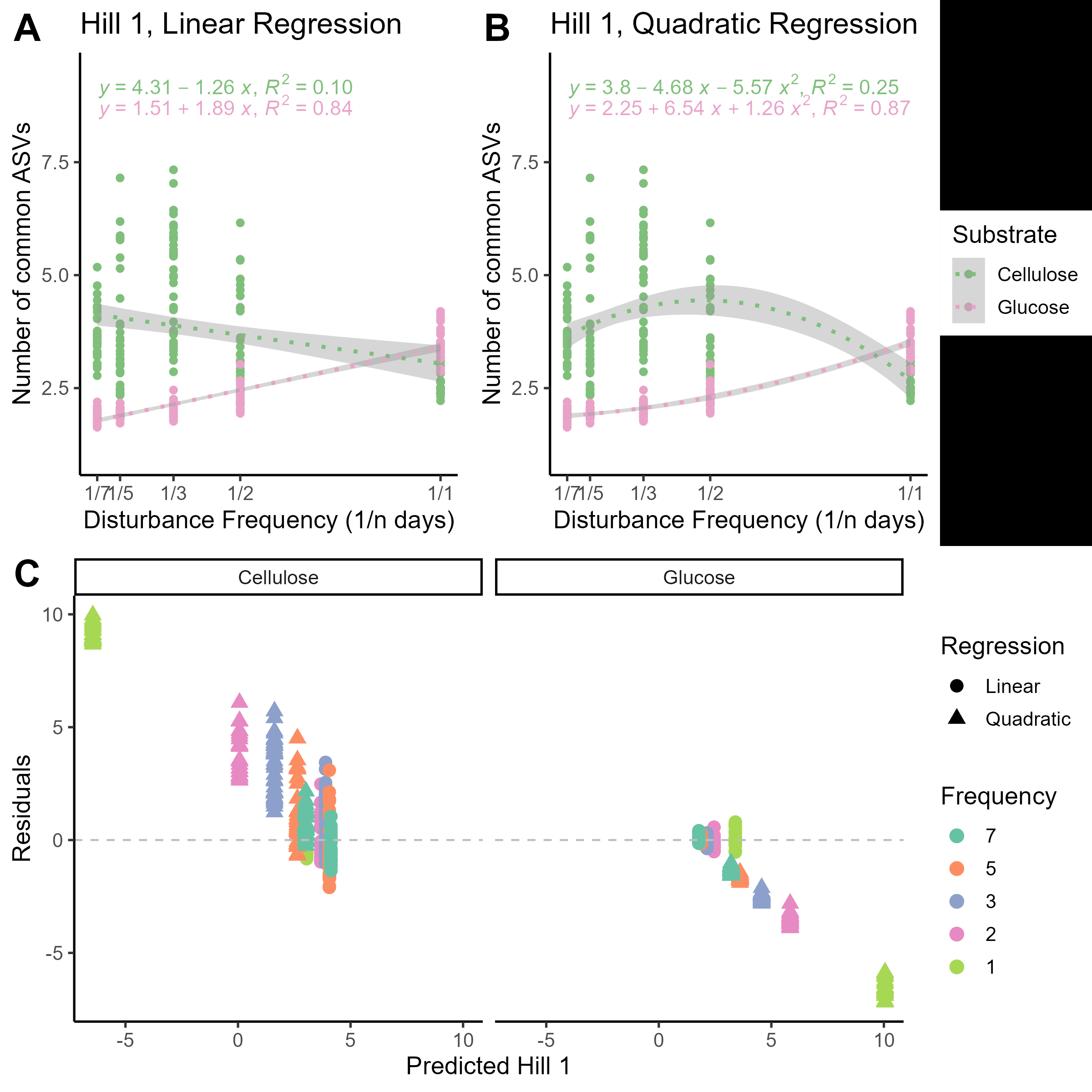

Supplement: Figure S4 — A revisualization of Fig. 4. [file msystems.00887-23-s0005.tiff]

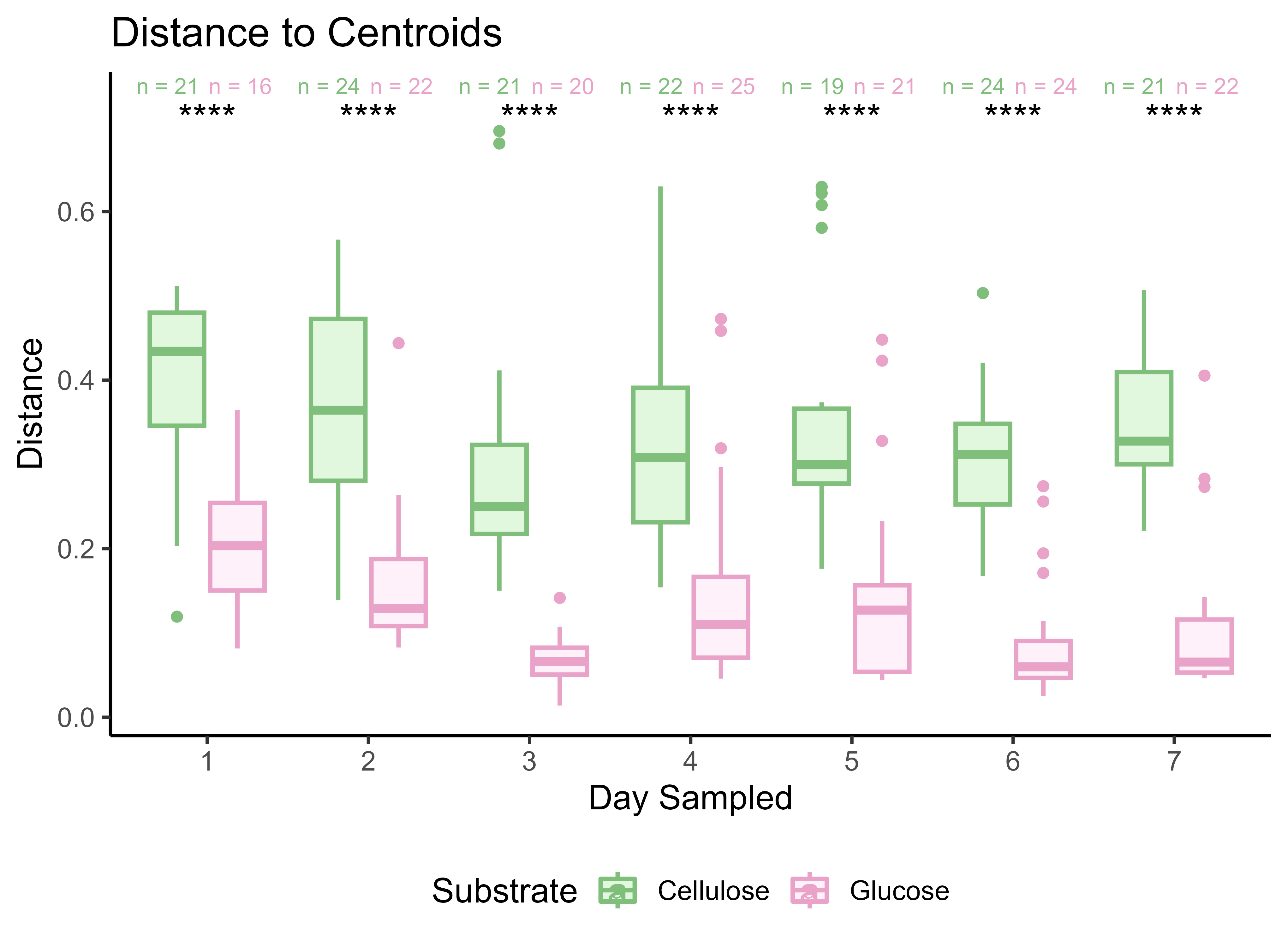

Supplement: Figure S5 — Boxplot of distance to centroids of samples. [file msystems.00887-23-s0006.tiff]
