# Supplementary material for: Disturbance–diversity relationships of microbial communities change based on growth substrate
Source: mSystems. 2024 Jan 23;9(2):e00887-23. doi: 10.1128/msystems.00887-23 (PMC10878081; doi:10.1128/msystems.00887-23)
Supplement: Supplemental legends — Legends for supplemental files [file msystems.00887-23-s0007.docx]

**Supplemental Figure 1.** Bar graph of relative abundance plot of samples from the disturbance frequency 1/2 treatment based on relative abundance of 16S rRNA gene amplicon sequencing of the top 15 most abundant ASVs and their assigned bacterial genus. The left graph represents samples grown on cellulose, and the right graph represents samples grown on glucose. Within each graph, samples are grouped by time sampled. Samples from day 5 of the cellulose treatment were removed from analysis, as we suspect they were mislabeled or mis-pipetted at some point prior to sequencing.

**Supplemental Figure 2.** Boxplot if Hill 0 and Hill 2 diversities. Green boxes represent samples grown on cellulose, pink boxes represent samples grown on glucose, and “n” refers to the number of samples within that disturbance frequency treatment. Significance values represent student’s t-test comparing cellulose samples to glucose samples of the same disturbance frequency treatment.

**Supplemental Figure 3.** A view of a 3D NMDS plot of community composition. Distance matrix was calculated using Bray-Curtis distance method. Communities grown in cellulose are shown as circles, and communities grown in glucose are shown as squares. Disturbance frequency is marked by color.

**Supplemental Figure 4.** A revisualization of Figure 4, where each point represents an individual sample, and the color of that point represents its substrate treatment. (A) has a linear regression, while (B) has a quadratic regression. The models were calculated from Hill 1 diversity measurements. (C) Residuals of linear and quadratic models (marked by a circle or triangle respectively) calculated from either cellulose or glucose samples. The color of points represent disturbance frequency treatments.

**Supplemental Figure 5.** Boxplot of distance to centroids of samples, clustered based on time sampled and grouped by substrate. Significance labels represent student’s t-test, comparing the mean distance between cellulose and glucose treatments of the same sampling time. The mean distance between sampling time for cellulose samples did not different significantly (ANOVA p-value = 0.085), and was statistically significant for glucose samples (ANOVA p-value = 2.6e-05).

**Supplemental Dataset 1.** NCBI accession numbers and metadata of 16S rRNA gene sequencing data generated for this work.
